# Computational modeling of residual stress in welded high-strength steel box sections

**DOI:** 10.1371/journal.pone.0332445

**Published:** 2025-09-16

**Authors:** Yihan Tu, Zhongming Liu, Yangsen Tu, Mingqiang Sheng

**Affiliations:** 1 School of Information Engineering, Gandong University, Fuzhou, China; 2 Academic Affairs Office, Nanchang Normal College of Applied Technology, Nanchang, China; 3 School of Engineering and Construction, Nanchang University, Nanchang, China; Islamic Azad University Mashhad Branch, IRAN, ISLAMIC REPUBLIC OF

## Abstract

This study investigates the residual stress patterns of welded box-section members constructed from high-strength steel (HSS). A finite element method (FEM) model developed in ANSYS is validated using experimental data from previous studies. Additionally, experimental data are directly utilized in the analysis to reinforce and contextualize numerical outcomes. A comprehensive parametric analysis explores the impact of plate thickness, width-to-thickness ratio, steel strength, welding sequence, and welding conditions on residual stress distributions. The results reveal that tensile residual stresses near weld regions consistently reach 82.6–97.8% of the yield strength and primarily depend on steel strength, with minimal sensitivity to section dimensions. In contrast, compressive residual stresses in mid-panel regions decrease by up to 72.2% with an increase in width-to-thickness ratio from 3.0 to 23.0, and the reduction rate is influenced by plate thickness. Additionally, welding sequences significantly affect residual stress magnitudes without altering their general distribution patterns. Diagonal welding method in the same direction effectively reduces mid-panel compressive stresses by up to 17.0%, and butt welds generate approximately 48.3% lower residual stresses than fillet welds. A residual stress distribution model for HSS welded box sections is developed. The model shows good agreement with experimental data with average deviation within 9.5% and can serve as a simplified yet reliable input for structural design, safety assessment, and advanced finite element modeling of welded steel members.

## 1. Introduction

High-strength steel (HSS) has gained increasing attention in civil engineering due to its superior strength-to-weight ratio, enabling more efficient and lightweight structural designs. Among various structural forms, welded box-section members are extensively utilized in bridges, high-rise buildings, and offshore structures owing to their excellent load-bearing capacity and torsional rigidity [1]. However, welding-induced residual stresses and their impact on structural performance remain critical concerns, particularly in thin-walled and HSS sections [2,3].

Prior research has enhanced insight into residual stress distribution in welded HSS sections, focusing primarily on geometric and material influences. Su et al. [4] observed that the ratio of compressive residual stresses to yield strength in ultra-HSS welded sections is lower than that in normal strength steel (NSS). Ban et al. [5] and Nie et al. [6] developed residual stress distribution models for 460 MPa welded box sections, emphasizing geometric parameters like width-to-thickness ratios. Wang et al. [7] provided further empirical support, revealing relatively lower residual stress ratios in Q460 HSS box-section. Additionally, Somodi and Kovesdi [8] and Rasmussen and Hancock [9] provided comprehensive regional insights through residual stress measurements on 690 MPa welded box sections. Cao et al. [10] systematically analyzed the impact of width-to-thickness ratios in welded channel sections, reinforcing the significance of geometric factors in residual stress formation.

**Fig 1:**
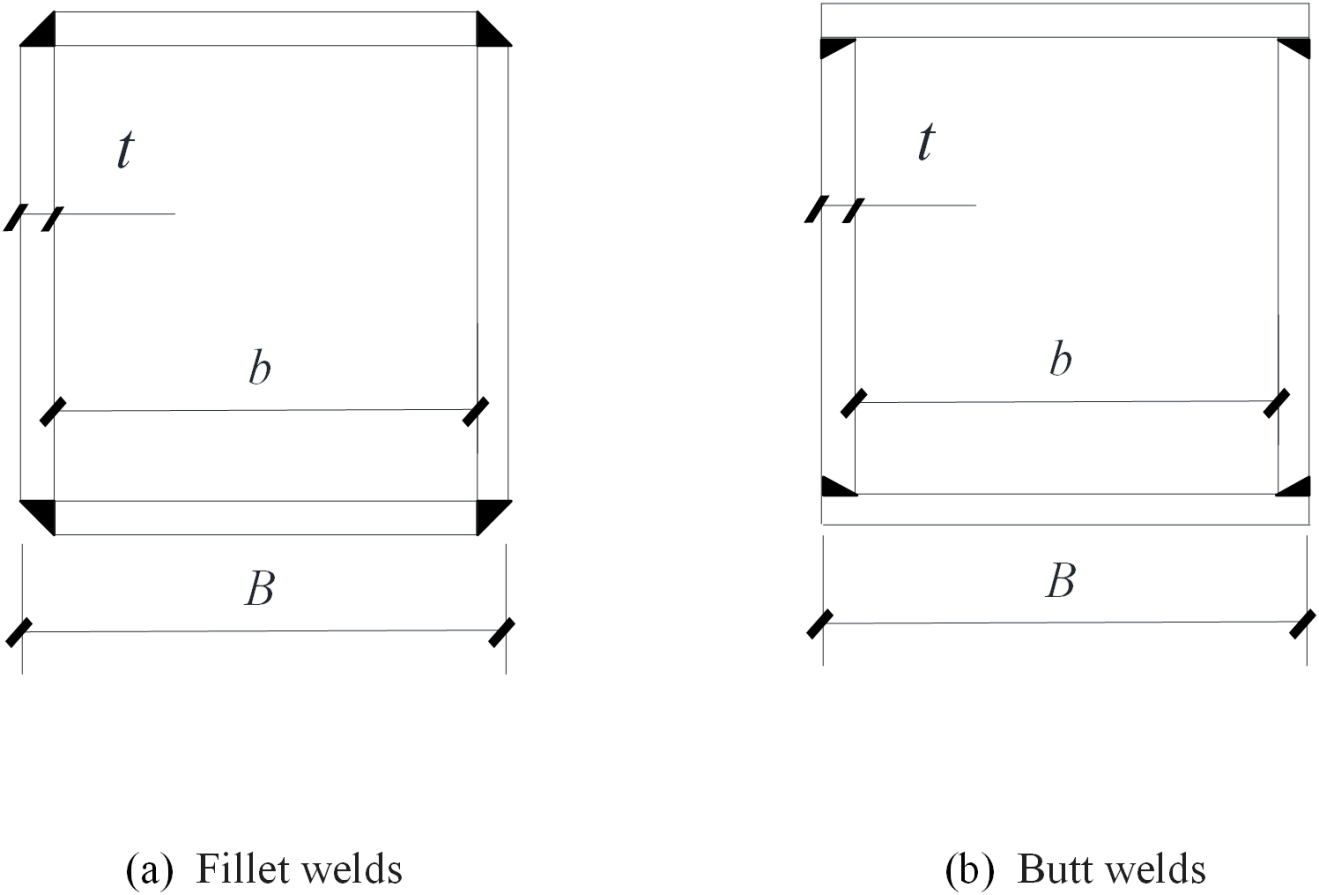
Weld types for box-section members.

Beyond geometric factors, welding techniques significantly influence residual stress formation [11]. Khan et al. [12] highlighted differences between fillet welds (Fig 1a) and butt welds (Fig 1b), underscoring the substantial impact of weld types and sequences on residual stress distribution. Gery et al. [13] and Chen et al. [14] studied the influence of welding sequence using the double ellipsoidal heat source model, demonstrating that welding sequences significantly impact residual stress distribution. Liu et al. [15] used ABAQUS to analyze residual stresses in welded H-sections fabricated from S355 and S690 steels, highlighting that multi-pass welding can significantly reduce tensile residual stresses (by 27%) and compressive stresses in the mid-panel (by up to 45%) compared to single-pass welding. Ghafouri et al. [16] examined the residual stress and deformation in welded T-joints made from S700 steel, concluding that mechanical boundary conditions have a greater influence on angular distortion and transverse residual stresses compared to welding sequences, whereas residual stresses show less sensitive to boundary constraints. Horváth et al. [17] further provided a comprehensive analysis of residual stresses in normal-strength steel (S355MC), HSS (S700MC), and hybrid members with single-bevel butt welds, summarized in Table 1.

**Table 1:** Summary of residual stresses in NSS and HSS.

Publication	B*H(mm^2^)	Thickness (mm)	*f*_*y*_ (MPa)	Weld type	Measurement technique	Weld details
Ban et al. [5]	100	10.0	RB1–460	a) butt weld	Sectioning technique	Single pass
	140	14.0	RB2–460			
	150	10.0	RB3–460			
	240	12.0	RB4–460			
	330	12.0	RB5–460			
	380	10.0	RB6–460			
Wang et al. [7]	88	11.0	Q460	a) butt weld	Sectioning technique	Single pass
	134	11.0	Q460			
	197	11.0	Q460			
Somodi and Kovesdi [8]	80	5.0	S355	a) butt weld	Sectioning technique	Single pass
	120	6.0	S355			
	150	6.0	S355			
	80	5.0	S460			
	120	6.0	S460			
	150	6.0	S460			
Khan et al. [12]	75	5.0	690	b) fillet weld	Non-destructive neutron diffraction technique	Single pass
	100	5.0	690			Single pass
	125	5.0	690			Single pass
	200	5.0	690			Single pass
	240	16.0	690			Six passes
	400	16.0	690			Six passes

Despite significant progress, existing studies primarily focus on isolated steel grades and limited parameters, highlighting the need for comprehensive residual stress distribution covering various HSS grades and welding scenarios. Therefore, this study aims to integrates experimental data [5] with FEM simulations to systematically analyze welding-induced residual stresses in HSS welded box-section members. A parametric study is carried out to examine the influence of critical variables such as width-to-thickness ratio, plate thickness, steel strength, welding sequence, and welding conditions on residual stress distribution. This study proposes a generalized, reproducible residual stress distribution model applicable across diverse HSS grades and geometries. Furthermore, this work employs a thermo-mechanically coupled FEM approach incorporating sequential welding simulations, significantly expanding the modeling detail and practical applicability. This comprehensive framework is designed to provide clear, adaptable guidance for engineering practice, ensuring robust and efficient design of HSS welded structures.

## 2 Experimental reference

**Fig 2:**
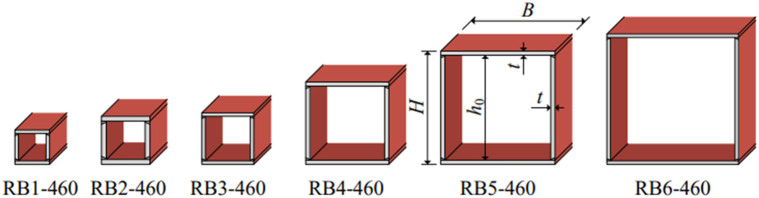
Schematic diagram of specimens [5].

Ban et al. [5] conducted an experimental study to examine the residual stress distribution in six welded box-section specimens constructed from 460 MPa HSS. Table 2 presents the detailed geometrical parameters of the specimens. The flange and web are jointed using single-bevel full-penetration butt welds with single-pass welding, as presented in Fig 2. The welding current ranged from 230 A to 235 A, with a voltage of approximately 25 V. The welding quality and width-to-thickness ratio of specimens complied with GB50205−2012 [18] and AWS ER120S-G [19].

**Table 2:** Geometrical parameters of specimens [5].

Specimens ID	*H* (mm)	*B* (mm)	*t* (mm)	*h*_*0*_ */t*
RB1–460	100	100	10	8.0
RB2–460	140	140	14	8.0
RB3–460	150	150	10	13.0
RB4–460	240	240	12	18.0
RB5–460	330	330	12	25.5
RB6–460	380	380	10	36.0

**Fig 3:**
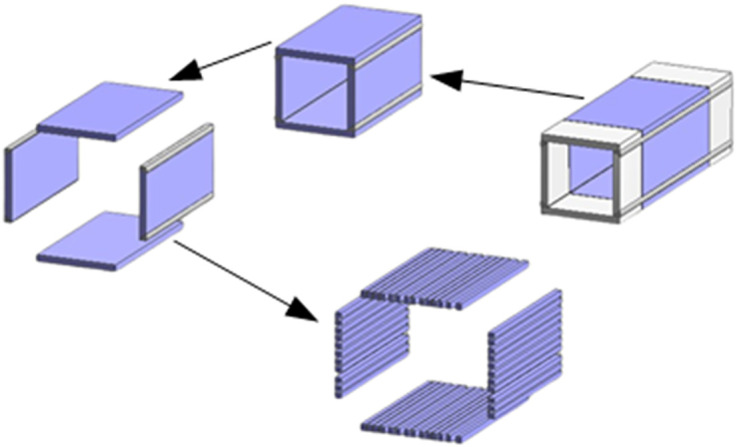
Sectioning method.


The residual stresses of the specimens are obtained through the sectioning method, as shown in Fig 3. Sections for stress measurement are extracted from the mid-length of each original member. Each section has a length at least three times greater than its larger cross-sectional dimension, with its ends positioned 1.5 to 2.0 times that dimension away from the specimen ends. Each strip has a width of 10 mm. Deformation before and after sectioning are obtained using a Whittemore strain gauge. To minimize thermal effects, reference holes are drilled at both ends of each strip by cold-machining. Initial reference data and temperature are recorded before the sectioning process. Changes in hole spacing and strip deformation after sectioning are used to determine the released residual stresses using the following equations [5].

$$\sigma_{r}=-E\;\varepsilon_{r}$$ (1)

where


$$\varepsilon_{r}=\left\{ \begin{array}{c} \varepsilon_{0}-\varepsilon_{t}\;No\;obvious\;bending\\ \bar{\varepsilon }-\varepsilon_{t}\;Obvious\;bending \end{array}\right.$$ (2)

$$\varepsilon_{0}=\frac{\left(r_{0}+r_{2}\right)-\left(r_{0}+r_{1}\right)}{r_{0}+r_{1}}$$ (3)

$$\varepsilon_{t}=\frac{\left(r_{0}+r_{t2}\right)-\left(r_{0}+r_{t1}\right)}{r_{0}+r_{t1}}$$ (4)

$$\bar{\varepsilon }=\varepsilon_{0}+\frac{{\left(\frac{\delta }{l}\right)}^{2}}{6{\left(\frac{\delta }{l}\right)}^{4}+1}$$ (5)

where, *E* is elastic modulus of 460 MPa steel; *ε*_*r*_ represent the final residual strain; *ε*_*0*_ and *ε*_*t*_ are the strains of the sectioning and thermal compensation strips, respectively; *ε̅* represents the adjusted strain post-bending; *r*_*0*_ is 254 mm (the length of Whittemore strain gauge); *r*_*1*_ represents the original spacing of the two holes, while *r*_*2*_ denotes their spacing measured on the strips after full sectioning; *r*_*t1*_, *r*_*t2*_ are values recorded by the compensation strip for temperature before initial and after full sectioning sectioning; *l* represents the post-bending strip length; and *δ* is the mid-span offset after bending.

## 3 Numerical analysis

**Fig 4:**
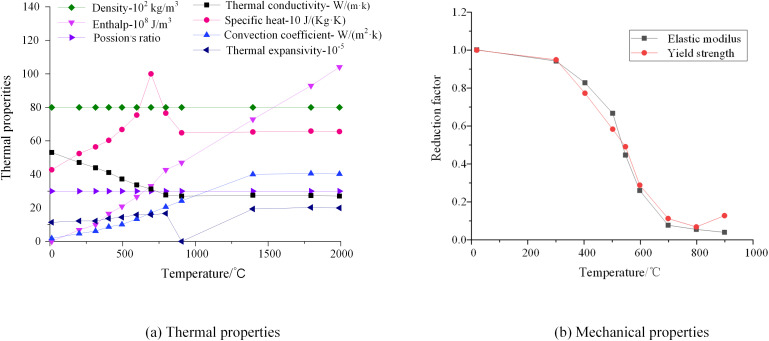
Properties of 460 MPa HSS.

A FEM of HSS welded box section is established in ANSYS to analyze the residual stress patterns. The analysis adopts an indirect evaluation method for welding-induced stresses and employs SOLID70 elements with thermo-structural coupling capabilities [15]. To simplify the calculations, the initial temperature of the component is specified as 20°C, with a welding speed of 10 mm/s. Based on the approach established by Cao et al. [20], the model adopts a constant heat input, neglects chemical reactions in the molten pool and material differences between the electrode and base metal, and considers only convective heat transfer with the surrounding air. These simplifications have been demonstrated to be sufficient for accurately predicting residual stress distributions in welded HSS sections. Fig 4 presents the thermodynamic parameters along with yield stresses and elastic modulus.

**Fig 5:**
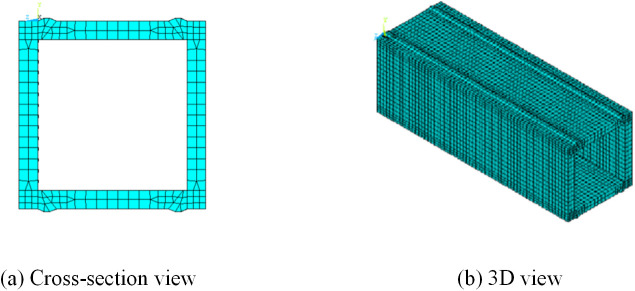
FEM of HSS welded box-section.

The mesh generation of the HSS welded box-section model is presented in Figs 5(a) and 5(b). Convective heat transfer coefficients, which vary with temperature and time, are applied to all surfaces exposed to air using interpolation tables. Boundary conditions include fully constraining one end in the *x*, *y*, and *z* directions, while the opposite end can freely move in the *z* direction but remains constrained in the *x* and *y* directions [15].

As noted by Tofangi et al. [21] and Nemati et al. [22], thermal behavior plays a important role in the performance of steel. To capture the thermal effects during welding, the heat generation rate is applied as a thermal load to simulate welding-induced thermal stresses in the full-penetration groove butt weld. The birth-death element method in ANSYS is used to model the sequential activation of weld bead filling. Computational convergence and stability are maintained using the Full Newton-Raphson method in conjunction with automatic time-stepping. The heat generation rate within the weld elements is determined using Eq. (6) [17].


$$q=\frac{\eta UI}{A_{weld}\times v\times dt}$$ (6)

Where *η* is the thermal efficiency (set to 0.7); *U* is welding voltage (25V); *I* is welding current (235A); *A*_*weld*_ denotes the weld cross-sectional area; v is the welding speed (10 mm/s), and *dt* is the time increment for each load step.

**Fig 6:**
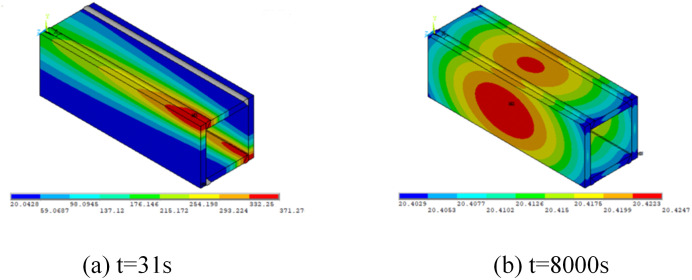
Temperature distribution during welding.

The welding simulation consists of four welds performed in two stages. Each stage comprises two diagonally opposite welds, followed by a cooling interval allowing the structure to air-cool to approximately 100°C before initiating the subsequent welds. The final cooling phase returns the structure to room temperature (20°C). Fig 6 illustrates the temperature distribution at various welding stages, demonstrating rapid temperature increases during welding followed by gradual cooling. The cooling period between 31s and 240s represents the interval between completing the first weld group and initiating the second.

**Fig 7:**
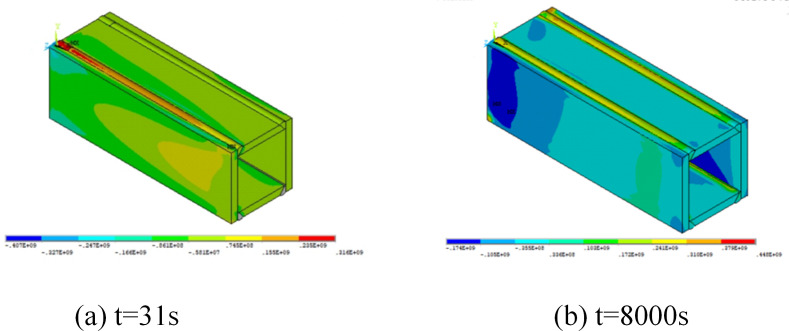
Residual stress distribution during welding.

The stress field analysis, derived from the temperature distribution results, is shown in Fig 7. Immediately after applying heat, the weld regions experience stresses around 360 MPa. During cooling, longitudinal tensile stresses develop due to thermal contraction constrained by surrounding material. Upon reaching room temperature, tensile stresses near the welds approach the yield strength, while the mid-regions of the flange plates experience compressive stresses. These compressive zones may reduce the local buckling resistance of thin-walled members, while tensile stress concentrations at the welds could serve as potential initiation points for yielding. Although failure modes are not directly analyzed in this study, the stress distribution provides valuable insight into possible failure-prone regions in practical applications.

## 4 Validation of FEM

**Fig 8:**
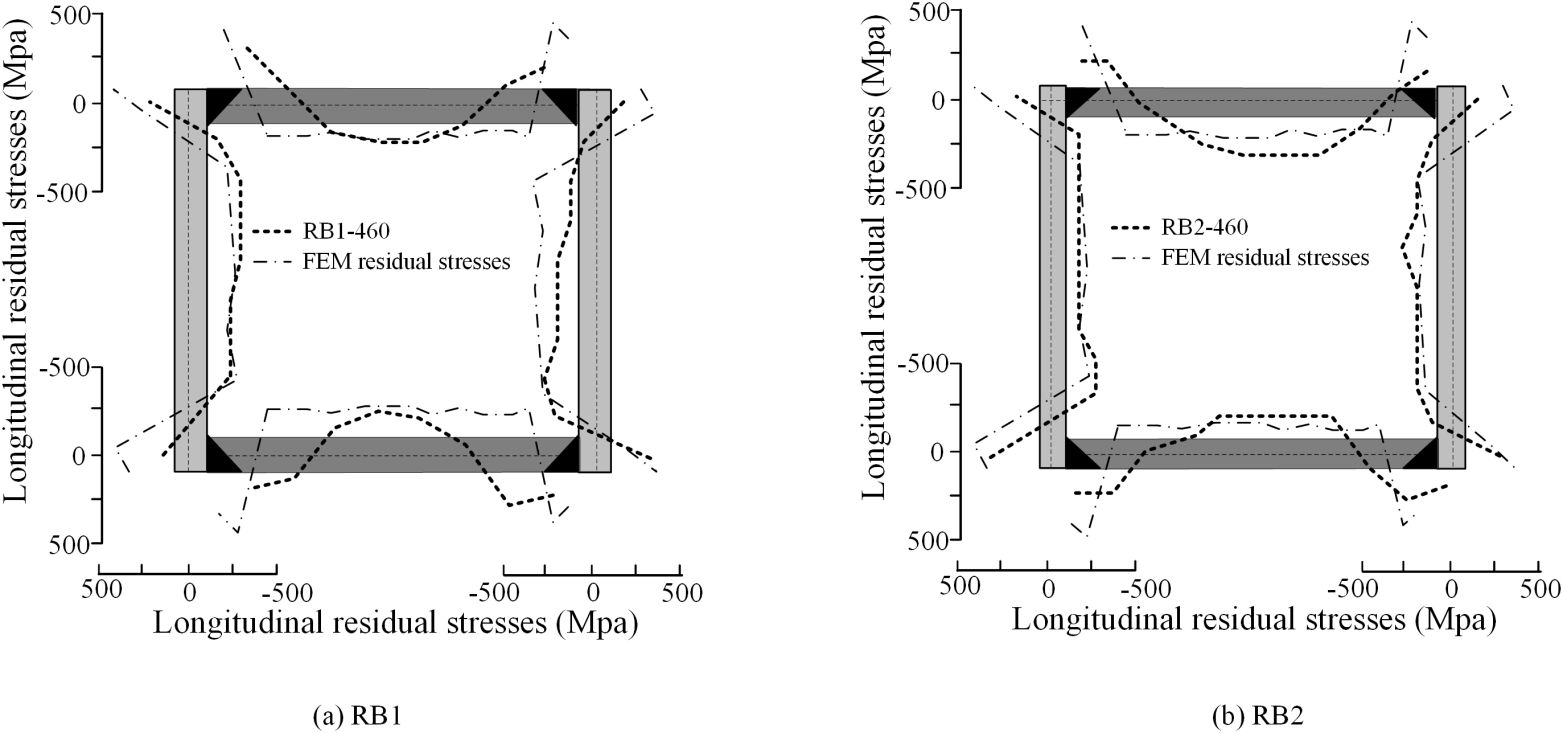
Comparison between experimental data and FEM results.

The six welded box-section specimens studied by Ban et al. [5], listed in Table 2, are numerically simulated using FEM. The average residual stresses at key locations are summarized in Table 3. The comparison of residual stress distribution between experimental and FEM results is presented in Fig 8. The results indicates that the FEM results exhibit a trend consistent with the experimental data, showing good overall agreement.

**Table 3:** Comparison of the residual stresses between test data and FEM results.

Specimen ID	*t*/*h*_*0*_	Test			FEM			Error^d^
		*σ*_*rc*_^a^	*σ*_*rt*_^b^	*L*_*c*_^c^ */B*	*σ*_*rc*_	*σ*_*rt*_	*L*_*c*_ */B*	*σ*_*rc*_
RB1–460	0.13	−185	201	0.60	−175	430	0.51	5.41%
RB2–460	0.13	−184	187	0.67	−163	427	0.55	11.41%
RB3–460	0.08	−164	318	0.69	−150	445	0.69	8.53%
RB4–460	0.06	−94	318	0.74	−90	450	0.71	4.25%
RB5–460	0.04	−69	329	0.80	−84	430	0.78	21.74%
RB6–460	0.03	−79	299	0.88	−73	440	0.80	7.60%
Average	–	–	–	–	–	–	–	9.82%
R^2^	–	–	–	–	–	–	–	0.93

^a^*σ*_*rc*_ and ^b^*σ*_*rt*_ represent the average compressive residual stresses in the mid-section of plates and the maximum tensile stresses in the weld region, respectively, ^c^*L*_*c*_ denotes the region of compressive residual stress distribution, and the ^d^Error = (Test data-FEM results)/ (Test data) × 100%.

A comparison of tensile residual stresses in weld region shows that experimental values remain below the yield strength, whereas the FEM accurately predicts the maximum tensile stresses (Table 3). This discrepancy may be attributed to partial residual stresses loss due to specimen impact or handling during testing. Additionally, a comparison of average compressive residual stresses in mid-section of plates shows a 13% difference between the experimental and FEM results for specimen B2, while specimen B4 demonstrates excellent agreement with only a 4% difference. On average, the discrepancy between the experimental data and FEM results across all specimens is 9.82%, with the model explaining 93.1% of the variance in the experimental data (i.e., R^2^ = 0.931).

Furthermore, a comparison of the compressive stress distribution region (*L*_*c*_/*B*) between experimental and FEM results shows that FEM consistently predicts a larger compressive stress distribution area, as shown in Table 3. This discrepancy arises because FEM simulations always achieve yield strength in the weld region, exceeding the experimentally measured values. This observation aligns with the self-equilibrium condition of the plate [23]. Overall, the FEM predictions agree well with the experimental data.

## 5 Parametric studies

A total of 29 models are developed to study residual stress distribution in HSS welded box sections, as summarized in Table 4. The models cover plate thicknesses of 5–10 mm, width-to-thickness ratios of 3–23, and steel grades of 460–960 MPa, ensuring broad engineering relevance.

**Table 4:** Parametric studies.

Specimen ID	*f*_*y*_ (MPa)	*B*/mm	*H*/mm	*t*/mm	*h*_*0*_ */t*	*t*/*h*_*0*_	*σ*_*rc*_ (MPa)	*σ*_*rc*_ */f*_*y*_	*σ*_*rt*_ (MPa)	*σ*_*rt*_ */f*_*y*_	*L*_*c*_ */B*
B1	460	50	50	10	3	0.33	−250	−0.54	430	0.93	0.31
B2	460	70	70	10	5	0.20	−223	−0.48	415	0.90	0.43
B3	460	80	80	10	6	0.17	−200	−0.43	380	0.83	0.51
B4	460	90	90	10	7	0.14	−180	−0.39	415	0.90	0.60
B5	460	100	100	10	8	0.13	−157	−0.36	400	0.87	0.55
B6	460	120	120	10	10	0.10	−139	−0.30	430	0.93	0.63
B7	460	170	170	10	15	0.07	−110	−0.22	427	0.93	0.71
B8	460	110	110	10	9	0.11	−140	−0.30	420	0.91	0.58
B9	460	130	130	10	13	0.08	−128	−0.28	419	0.91	0.73
B10	460	150	150	10	17	0.06	−116	−0.25	420	0.91	0.67
B11	460	190	190	10	21	0.05	−87	−0.19	419	0.91	0.77
B12	460	210	210	10	25	0.04	−86	−0.19	437	0.95	0.73
B13	460	230	230	10	30	0.03	−75	−0.16	428	0.93	0.75
B14	460	250	250	10	36	0.03	−70	−0.15	450	0.98	0.80
B15	550	100	100	10	8	0.13	−165	−0.29	490	0.89	0.45
B16	690	100	100	10	8	0.13	−152	−0.21	600	0.87	0.55
B17	800	100	100	10	8	0.13	−173	−0.22	690	0.86	0.43
B18	960	100	100	10	8	0.13	−176	−0.17	860	0.90	0.45
B19	550	70	70	7	8	0.13	−160	−0.30	490	0.88	0.50
B20	690	70	70	7	8	0.13	−145	−0.22	598	0.87	0.49
B21	800	70	70	7	8	0.13	−149	−0.19	730	0.91	0.54
B22	960	70	70	7	8	0.13	−165	−0.18	840	0.88	0.57
B23	460	50	50	5	8	0.13	−175	−0.38	435	0.95	0.53
B24	460	60	60	5	10	0.10	−138	−0.31	400	0.87	0.69
B25	460	85	85	5	15	0.07	−96	−0.23	395	0.86	0.75
B26	460	70	70	7	8	0.13	−165	−0.34	389	0.85	0.56
B27	460	84	84	7	10	0.10	−145	−0.32	413	0.90	0.60
B28	460	119	119	7	15	0.07	−100	−0.22	450	0.98	0.65
B29	690	80	80	5	16	0.06	−105	−0.15	600	0.87	0.69

The impact of welding sequence on residual stress distribution is further analyzed through a comparative study of three distinct welding techniques. Additionally, Specimen B29 (steel grade 690 MPa, plate thickness 5 mm, width-to-thickness ratio 8) is compared with findings from Khan et al. [12] to assess the effect of different welding types on residual stress distributions in HSS welded box sections.

### 5.1 Section dimensions

**Fig 9:**
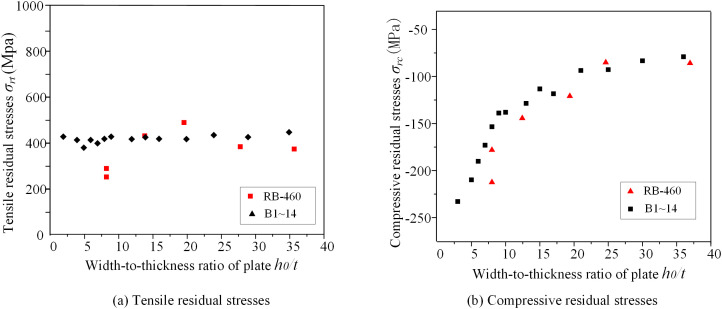
Residual stresses versus the width-to-thickness ratio.

A summary of experimental data and FEM results for welded box sections made from 460 MPa steel with a plate thickness of 10 mm is conducted to analyze how width-to-thickness ratios influence residual stress patterns in the weld zone, as presented in Figs 9(a) and 9(b). The findings show that tensile residual stresses near the weld consistently approach the yield strength (*f*_*y*_) and exhibit no clear relationship with the width-to-thickness ratio. In contrast, compressive residual stresses (*σ*_*rc*_) at the middle of plates declines as the width-to-thickness ratio rises, though the rate of decrease becomes less pronounced beyond a certain threshold. For example, when the width-to-thickness ratio increase from 3.00 to 23.00, *σ*_*rc1*_ decreases from −0.54 *f*_*y*_ to −0.15 *f*_*y*_, as presented in Fig 9(b).

**Fig 10:**
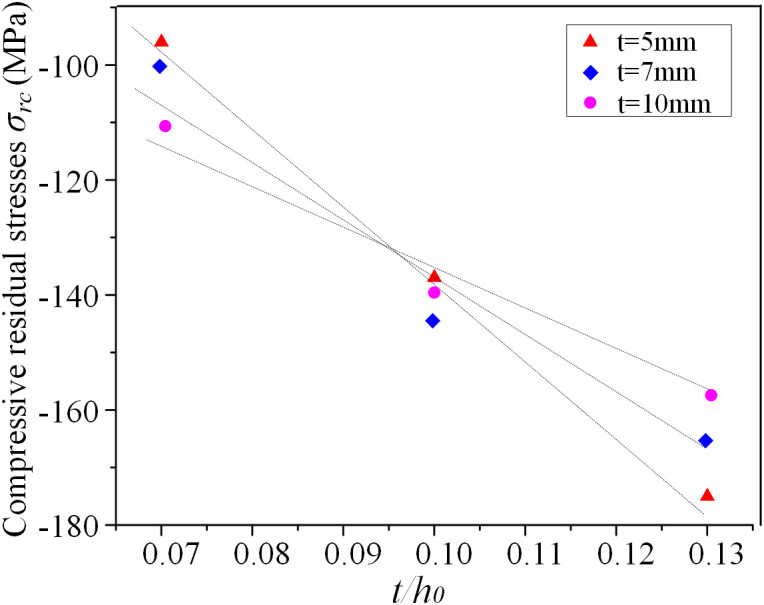
Magnitude of compressive residual stresses versus the *t/h*_*0*._

Specimens with thicknesses of 5, 7, and 10 mm are studied to evaluate the impact of plate thickness on compressive residual stresses, under varying width-to-thickness ratios, as shown in Fig 10. The results reveal that for thinner plates, the compressive residual stresses exhibit strong sensitivity to the width-to-thickness ratio, while this sensitivity decreases gradually as plate thickness increases. For instance, when the plate thickness is 5 mm, an increase in *t*/*h*_0_ from 0.07 to 0.13 leads to a 45% reduction in the compressive residual stresses, whereas for a 10 mm plate, the reduction is only 30%.

**Fig 11:**
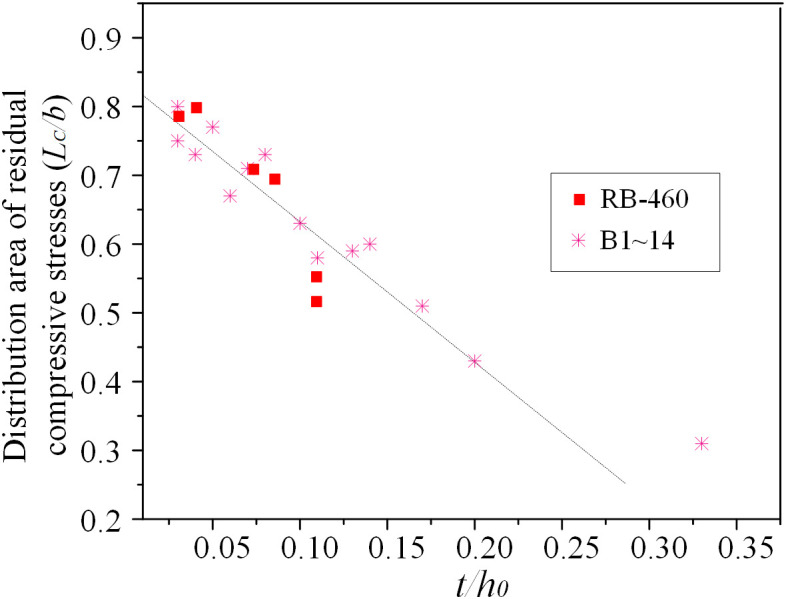
Distribution region of compressive residual stresses versus the *t/h*_*0*._

Fig 11 illustrates how the residual stress distribution region varies with the ratio *t*/*h*_0_. The results show that when the plate thickness remains constant, an increase in the *t*/*h*_0_ leads to an expanded region of compressive residual stress distribution relative to the plate width.

### 5.2 Steel strength

**Fig 12:**
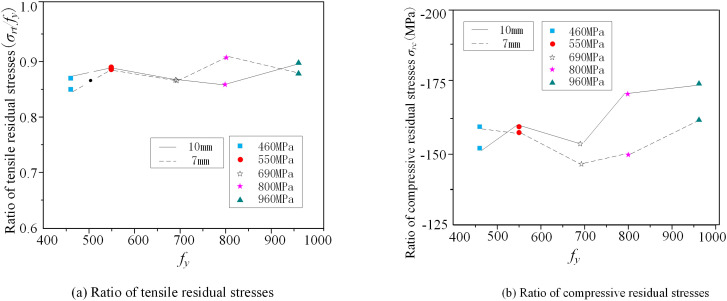
Residual stresses versus steel strength.

Fig 12(a) presents the correlation between tensile residual stress coefficient and various strength grades of box sections. The results indicate that for both 7 mm and 10 mm plate thicknesses, tensile stresses near the weld consistently reach 82.6–97.8% of the yield strength of the material, showing no significant correlation with plate thickness. In contrast, compressive residual stresses show stronger dependence on the width-to-thickness ratio than on steel strength. A comparison between 7 mm and 10 mm plates shows that the thicker (10 mm) plates experience higher average compressive stresses in the mid-panel region. This occurs because thicker plates undergo greater temperature gradients between their inner and outer surfaces during cooling, resulting in larger differential shrinkage. Consequently, the compressive residual stress distribution in thicker plates becomes less uniform (Fig 12(b)), thereby increasing the compressive stresses in the central region of plate. Similar results are found by Wang et al. [24].

### 5.3 Welding condition

**Fig 13:**
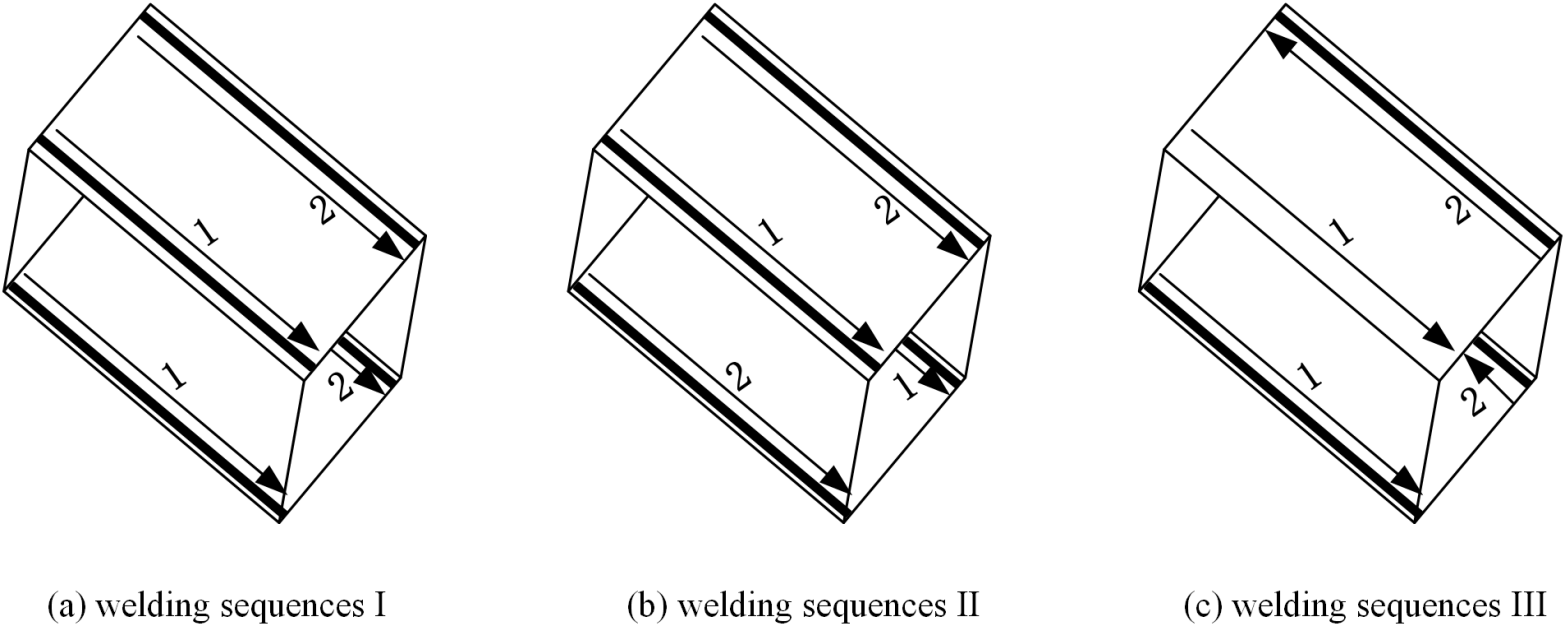
Different welding sequences.

Chen et al. [14] found that welding sequence has a significant impact on residual stresses. Therefore, to investigate its effect in HSS welded box sections, three different welding sequences are applied to Specimen B1, as presented in Fig 13.

**Fig 14:**
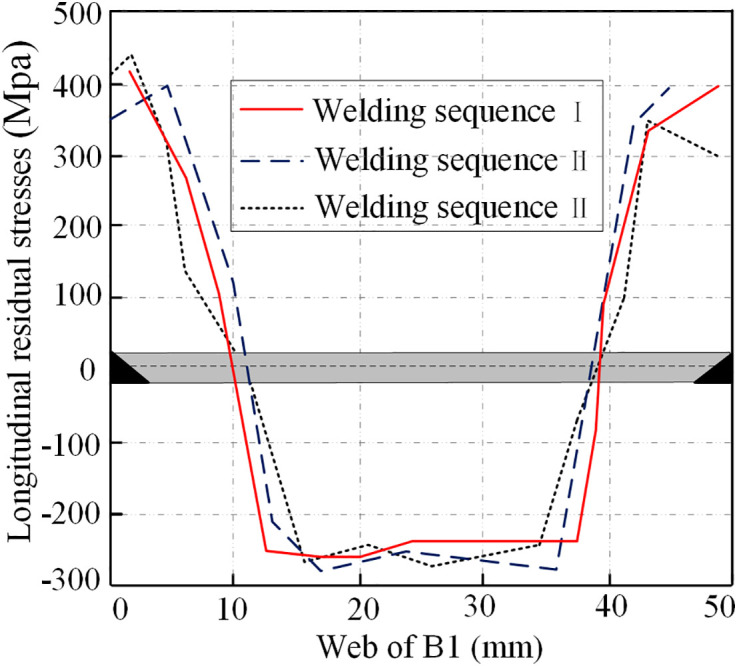
Comparison of longitudinal residual stresses among different welding sequences.

The residual stress distribution of three welding sequences is compared, as shown in Fig 14. Although different welding sequences do not significantly alter the overall residual stress distribution patterns, they significantly affect the magnitude of residual stresses. In each sequence, the tensile residual stress at the weld reaches the yield strength of materials. The average compressive residual stress in the mid-panel follows the order: sequence I < sequence III < sequence II, with sequence I showing reductions of 10% and 17% compared to sequences III and II, respectively. Consequently, the diagonal welding method in the same direction (sequence I) emerges as the most effective approach, aligning better with practical engineering applications.

**Fig 15:**
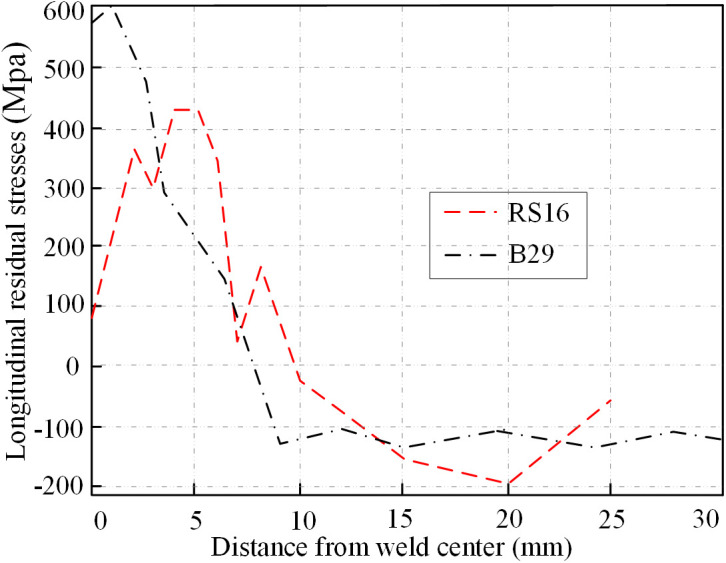
Comparison of residual stresses between RS16 and B29.

Khan et al. [12] conducted residual stress measurements on the Q690 box section (Specimen RS16) using a non-destructive neutron diffraction technique. The specimen was welded using single-pass fillet welds, as illustrated in Fig 1(b). The peak tensile stress near the weld reaches 0.72 *f*_*y*_, the average compressive stress is −0.29 *f*_*y*_, and the compressive stress region in the mid-panel covers approximately 29% of the section width. A comparison of these experimental results with FEM outcomes for Specimen B29 (butt-welded) reveals that the average compressive residual stresses in the mid-panel region is −0.15 *f*_*y*_, lower than that observed in Specimen RS16, as depicted in Fig 15. This difference suggests that butt-welded sections tend to exhibit lower mid-panel compressive residual stresses compared to fillet-welded sections.

## 6 Distribution models of residual stresses

Based on experimental observations and FEM analysis, a multi-segment residual stress model for HSS welded box-section members is proposed. This model ensures self-equilibrium of axial forces and bending moments while simplifying the analysis. As illustrated in Fig 8, peak tensile stresses occur in the weld regions, whereas compressive stresses are concentrated at the mid-width of the plate, and there is a linear transition between these regions.

### 6.1 The magnitudes of residual stresses

According to experimental and FEM results, tensile residual stresses are positively correlated with steel strength but shows no significant relationship with the width-to-thickness ratio or plate thickness. For HSS welded box sections, the tensile residual stresses in the weld region are conservatively assumed to be equal to the yield strength (*f*_*y*_), encompassing 95% of experimental and FEM data [25,26].

Compressive residual stresses are independent of steel strength but varies notably with the width-to-thickness ratio and steel thickness. Accordingly, Eq. (7) is used to estimate the compressive residual stresses (*σ*_*rc*_), which range from −460 MPa to 46 MPa.


$$\sigma_{rc}=-68-\frac{675}{b/t}-\frac{11}{t}$$ (7)

### 6.2 The parameters for distribution zone

Ban et al. [5] and Somodi and Kövesdi [8] found that the widths of the tensile and compressive zones are influenced by steel strength and plate thickness, as listed in Table 5. In this study, for HSS welded box sections, the parameters defining the distribution zones of HSS welded box sections are determined using Eqs. (8) – (12).

**Table 5:** Width of the tension region.

Steel material	Parameter	Recommended value
460MPa [5]	*a*	*h*_*o*_/20
S235 [8]	*a*	2.5*t*
S355-S460 [8]	*a*	2*t*
S500 [8]	*a*	1.5*t*
S700 [8]	*a*	0.75*t*
S900 [8]	*a*	0


$$\int \;\;{\int \sigma }_{fr}\;.\;dA=0$$ (8)

a+b+c+d+e=B (9)

f+b+c+d+g=H (10)

f+t=a (11)

g+t=e (12)

Where *A* represents the cross-sectional area, *σ*_*fr*_ is the residual stresses on the plate, *a*, *b*, *c*, *d*, *e*, *f*, and *g* are the distribution region parameters, and *t* denotes the plate thickness.

The widths of tensile zones (parameters *a* and *e*) are defined as 0.1*h*_*0*_. The remaining zone parameters (*b*, *c*, and *d*) are calculated by solving the geometric and residual stresses self-equilibrium equations (Eqs. (8)–(10)), as presented in Table 6.

**Table 6:** Suggested values for parameters in proposed residual stress distribution model.

Parameters	*a*	*b*	*c*	*d*
Suggested values	0.1*h*_*o*_	Eqs. (8)-(9)	Eqs. (8)-(9)	Eqs. (8)-(9)

**Fig 16:**
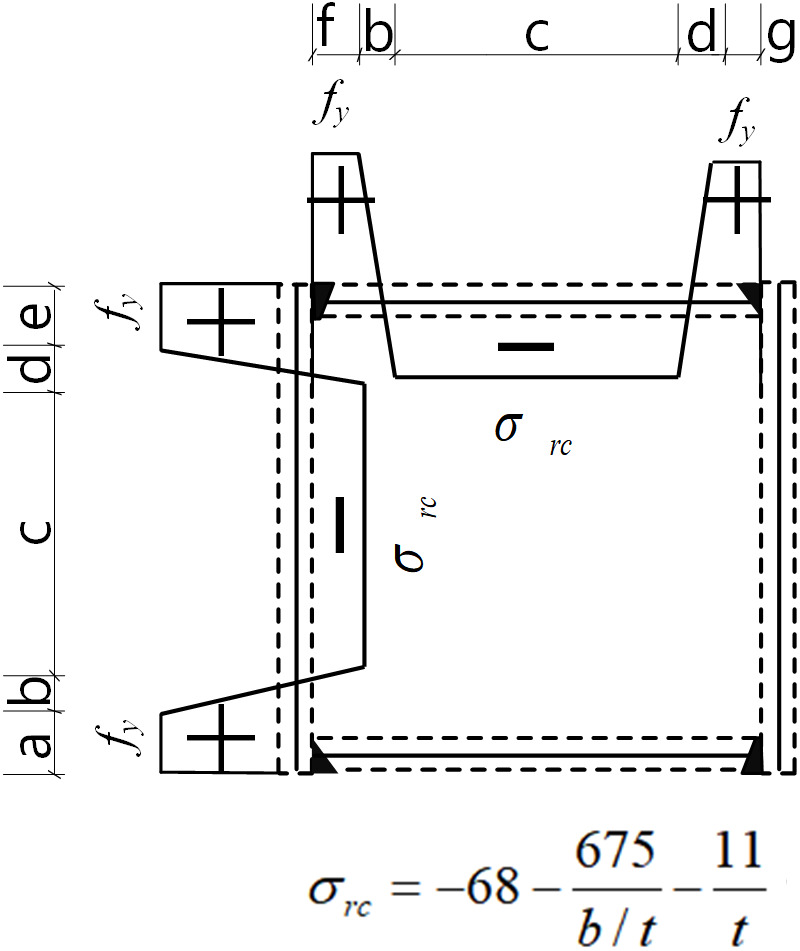
Residual stress distribution model for HSS welded box section.

**Fig 17:**
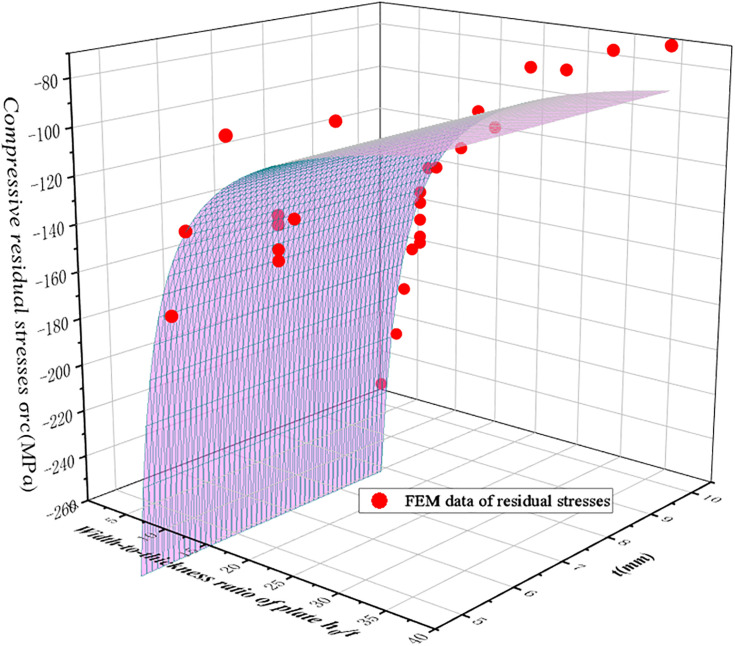
Comparison of residual stresses between the proposed model and results.

Based on these results, the finalized residual stress distribution model for HSS welded box sections is proposed, as depicted in Fig 16. The comparison between the proposed model and both experimental and FEM results is illustrated in Fig 17 and Table 7. The results indicate that the proposed model closely matches the experimental and FEM results, with average errors of −4.24% in compressive residual stress and −9.50% in tensile residual stress.

**Table 7:** Comparison of residual stress between the proposed model and test data.

Specimen ID	Test		Proposed model		Error	
	*σ*_*rc*_	*σ*_*rt*_	*σ*_*rc*_	*σ*_*rt*_	*σ*_*rc*_	*σ*_*rt*_
B1	−250	430	−294	460	−17.60%	−6.98%
B2	−223	415	−204	460	8.52%	−10.84%
B3	−200	380	−182	460	9.00%	−21.05%
B4	−180	415	−166	460	7.78%	−10.84%
B5	−157	400	−153	460	2.55%	−15.00%
B6	−139	430	−137	460	1.44%	−6.98%
B7	−110	427	−114	460	−3.64%	−7.73%
B8	−140	420	−144	460	−2.86%	−9.52%
B9	−128	419	−121	460	5.47%	−9.79%
B10	−116	420	−109	460	6.03%	−9.52%
B11	−87	419	−101	460	−16.09%	−9.79%
B12	−86	437	−96	460	−11.63%	−5.26%
B13	−75	428	−92	460	−22.67%	−7.48%
B14	−70	450	−88	460	−25.71%	−2.22%
Average	–	–	–	–	−4.24%	−9.50%
Standard deviation	–	–	–	–	11.76%	4.30%

## 7 Conclusion

This study employs FEM to investigate the impact of plate thickness, width-to-thickness ratio, steel strength, and welding conditions on the residual stress distribution in HSS welded box section members. Based on the analyses, a residual stress distribution model for HSS welded box section is proposed. The key findings are summarized below:

(1) Compressive residual stresses in the mid-section of the plate decreases as the width-to-thickness ratio rises. The rate of the decrease is influenced by the plate thickness, while the magnitude of the compressive residual stresses shows no correlation with steel strength.
(2) The maximum tensile residual stresses near the weld region consistently approach the yield strength of steel, independent of section dimensions.
(3) The residual stress magnitude is markedly influenced by welding parameters, whereas the distribution form remains consistent. Among the three welding methods, the diagonal weld technique proves most effective in reducing mid-panel compressive stresses.
(4) A comparison between butt welds and fillet welds reveals that fillet welds generally produce slightly higher residual stresses compared to butt welds.
(5) This residual stress distribution model of HSS welded box sections shows excellent agreement with experimental data and FEM results, confirming its accuracy and applicability.

Building on these findings, this study extends existing experimental work by incorporating sequential welding simulations and enhancing the applicability of the model across a wide range of HSS grades and geometries. The resulting model offers a practical and efficient means for engineers to estimate residual stress distributions, facilitating advanced structural analysis and design.

Limitations of this study include the use of a single experimental dataset for validation and the exclusive focus on hot-rolled sections. Future research may explore model calibration for cold-formed sections and higher-strength steels exceeding 960 MPa, as well as the incorporation of residual stress effects into structural stability assessments and design code development.

### Nomenclature

**Table:** 

*E*	Elastic modulus of steel	*r*_*t1*_ *, r*_*t2*_	Compensation readings before and after sectioning
*f*_*y*_	Yield strength of steel	*δ*	Mid-span offset after bending
*σ*	Residual stress of steel	*B*	Width of the welded plate section
*σ*_*rc*_	Average compressive residual stress in the mid-panel	*H*	Total height of specimen
*σ*_*rt*_	Maximum tensile residual stress in the weld region	*t*	Plate thickness
*ε*	Strain of steel	*h*_*0*_	Inner clear height of specimen
*ε*_*r*_	Residual strain after sectioning	*t/h*_*0*_	Plate thickness to section height ratio
*ε*_*0*_	Strain in sectioning strip before cutting	*L*_*c*_	Region of compressive residual stress distribution
*ε*_*t*_	Temperature compensation strain	*q*	Heat generation rate
*ε̅*	Modified strain after bending	*η*	Thermal efficiency (typically 0.7)
*r*_*0*_	Length of Whittemore strain gauge	*U*	Welding voltage
*r*_*1*_	Initial spacing between reference holes	*I*	Welding current
*r*_*2*_	Spacing between reference holes after sectioning	*A*_*w*_	Cross-sectional area of the weld
*a*,*b*,*c*,*d,e,f,g*	Parameters defining the residual stress distribution zones	*v*	Welding speed
*l*	Strip length after bending		

## Supporting information

S1 File Supplemental figures and datasets. This Excel file contains additional figures and data supporting the findings of the study.
(XLSX)
